# Synergistic regulation of *Rgs4* mRNA by HuR and miR-26/RISC in neurons

**DOI:** 10.1080/15476286.2020.1795409

**Published:** 2020-08-11

**Authors:** Janina Ehses, Sandra M. Fernández-Moya, Luise Schröger, Michael A. Kiebler

**Affiliations:** BioMedical Center, Medical Faculty, Ludwig Maximilians University of Munich, Martinsried, Germany

**Keywords:** Mirna, RBP, cooperative binding, mRNA stability, neuronal RNA, Rgs4, HuR, ELAVL1, miR-26

## Abstract

The negative regulator of G-protein signalling 4 (Rgs4) is linked to several neurologic diseases, *e.g*. schizophrenia, addiction, seizure and pain perception. Consequently, Rgs4 expression is tightly regulated, resulting in high mRNA and protein turnover. The post-transcriptional control of gene expression is mediated via RNA-binding proteins (RBPs) that interact with mRNAs in a combinatorial fashion. Here, we show that in neurons the RBP HuR reduces endogenous Rgs4 expression by destabilizing *Rgs4* mRNA. Interestingly, in smooth muscle cells, *Rgs4* is stabilized by HuR, indicating tissue-dependent differences in HuR function. Using *in vitro* RNA-based pulldown experiments, we identify the functional AU-rich element (ARE) within the *Rgs4* 3ʹ-UTR that is recognized and bound by HuR. Bioinformatic analysis uncovered that this ARE lies within a highly conserved area next to a miR-26 binding site. We find that the neuronal-enriched miR-26 negatively influences Rgs4 expression in neurons. Further, HuR and miR-26 act synergistically in fluorescent reporter assays. Together, our data suggest a regulatory mechanism, in which an RBP selectively destabilizes a target mRNA in cooperation with a miRNA and the RISC machinery.

## Introduction

The negative regulator of G-protein signalling 4 (Rgs4) plays an important role in synaptic plasticity as well as in many diseases of the nervous system, including schizophrenia, addiction, seizure, pain and neurodegenerative disorders [1–5]. Rgs4 encodes a GTPase-activating protein of the G protein-coupled receptor (GPCR) pathway, modulating receptor-mediated neuronal signalling at the synapse [5,6]. Both Rgs4 protein as well as *Rgs4* mRNA show a high turnover rate, suggesting extensive post-transcriptional and post-translational regulation [7,8]. In contrast to the protein level, where Rgs4 regulation has been studied extensively [5], knowledge about the regulation of *Rgs4* mRNA in neurons is scarce. Post-transcriptional gene regulation enables spatially and temporally fine-tuned protein production and is key in the nervous system, where targeted local protein synthesis at single synapses can take place [9,10]. The control of this process is likely to be mediated by the combinatory binding of sequence- or structure-specific RNA-binding proteins (RBPs) and microRNAs (miRNAs) [11] preferentially to the 3ʹ-untranslated region (3ʹ-UTR) of target mRNAs. miRNAs are small noncoding RNAs that complementary bind and repress target mRNAs by associating with Argonaute (Ago) proteins [11] and recruiting the RNA induced silencing complex (RISC). In neurons, Rgs4 is post-transcriptionally regulated by the double-stranded RBP Staufen2. Endogenous *Rgs4* mRNA is reduced upon silencing of Staufen2 both *in vitro* [12] and *in vivo* [13], suggesting a role of Staufen2 in the regulation of *Rgs4* mRNA levels. In addition, Stau2 regulates dendritic transport of an *Rgs4* 3ʹ-UTR reporter in primary hippocampal neurons [14].

While certain RBPs are enriched in nervous tissue, *e.g*. FMRP, Staufen2 or Pumillio2 [15], the vast majority of RBPs are ubiquitously expressed. HuR/ELAVL1 is a ubiquitously expressed RBP with a crucial role in the nervous system [16–19] as well as in muscle [7,20,21]. *Rgs4* mRNA is a physiological target of HuR [7]. Overexpression of Rgs4 can rescue vascular phenotypes observed in smooth muscle cells deficient for HuR [21]. In those cells, HuR stabilizes *Rgs4* mRNA [7]. HuR binds to transcripts containing AU-rich elements (AREs), thereby mainly stabilizing the mRNA. There are cases, however, where HuR exerts the opposite effect [22,23]. Depending on its mode of action, it might act in a cooperative or competitive manner. Competition for binding with miRNAs due to steric hindrance or RNA structure-mediated effects has been reported [22,24,25].

To evaluate the role of HuR in the regulation of *Rgs4* mRNA expression in neurons, we used RNA interference by expressing a short hairpin RNA (shRNA) against HuR. The resulting downregulation of endogenous HuR expression in mature neurons caused an upregulation of *Rgs4* mRNA. Fluorescent reporter assays and *in vitro* RNA affinity purification allowed us to define the binding sites for both miR-26 and HuR in the *Rgs4* 3ʹ-UTR, which are both located within the same predicted, highly conserved RNA hairpin structure. Detailed analysis of both factors allowed us to unravel a synergistic action of the RBP HuR together with the miR-26/RISC complex in the regulation of *Rgs4* mRNA in mature neurons. Our proposed mechanism highlights the fine-tuned interplay between *trans*-acting factors, *e.g*. the RBP and the miRNA/RISC, depending on the RNA target structure.

## Results

### HuR destabilizes Rgs4 mRNA

**Figure 1.**
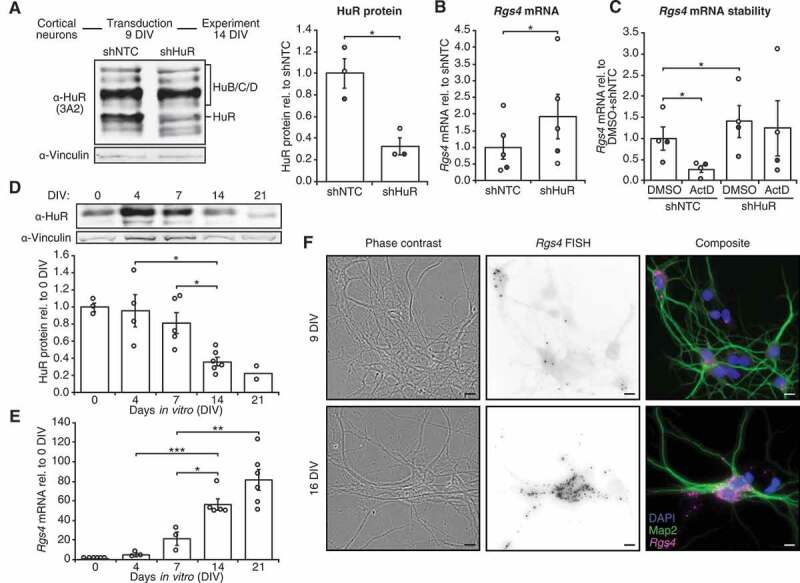
HuR destabilizes *Rgs4* mRNA in primary neurons. (A) Transduction of cortical neurons with shNTC or shHuR for 5 days. Left panel, experimental outline and Western blot against HuR of 14 DIV rat cortical neurons transduced at 9 + 5 DIV with lentiviruses expressing shNTC or shHuR. Right panel, quantification of HuR Western blot signal, normalized to shNTC. Paired Student’s *t*-test. (B) Quantification of endogenous *Rgs4* mRNA by qRT-PCR in 14 DIV cortical neurons transduced at 9 + 5 DIV with lentiviruses expressing shNTC or shHuR, normalized to shNTC. Paired Student’s *t*-test. (C) Analysis of *Rgs4* mRNA stability in 14 DIV cortical neurons transduced at 9 + 5 DIV with lentiviruses expressing shNTC or shHuR and treated with DMSO or ActD for 90 min at 14 DIV. *Rgs4* mRNA levels were quantified by qRT-PCR and normalized to DMSO+shNTC. Paired Student’s *t*-test. (D) Quantification of Western blot HuR protein signal of rat cortical neurons at different DIV, normalized to 0 DIV. Unpaired Student’s *t*-test. (E) Quantification of *Rgs4* mRNA qRT-PCR signal of rat hippocampal neurons at different DIV, normalized to 0 DIV. Unpaired Student’s *t*-test. (F) Representative phase-contrast and pseudo-coloured fluorescence images of cortical neurons at 9 DIV and 16 DIV showing *Rgs4* FISH signal (magenta), staining for Map2 (green) and DAPI (blue). Scale bar 10 µm. All error bars are SEM from ≥ 3 independent biological replicates; asterisks represent *p*-values (*p < 0.05, **p < 0.01, ***p < 0.001). NTC non-targeting control; ActD Actinomycin D; DIV *days in vitro*; FISH fluorescent *in situ* hybridization

We tested the influence of HuR expression on *Rgs4* mRNA levels in primary cortical neurons through knock-down (KD) of endogenous HuR by shRNA. The shRNA against HuR enables specific knock-down of HuR (Fig. 1A, Sup. Fig. 1B) but not of the neuron-specific Hu proteins, HuB/C/D (Sup. Fig. 1A). Contrary to published data from smooth muscle cells [7], *Rgs4* mRNA levels increased twofold upon knock-down of HuR (Fig. 1B). Downregulation of a different RBP, Pumilio2, did not result in altered *Rgs4* mRNA levels (Sup. Fig. 1C,D). We further determined if the *Rgs4* mRNA upregulation is due to effects of HuR on mRNA stability rather than transcription or splicing. As shown in Fig. 1C, treating neurons with the transcription inhibitor Actinomycin D resulted in a strong drop of *Rgs4* mRNA. This effect, however, could be rescued when HuR was knocked down (Fig. 1C, Sup. Fig. 1E). In order to rule out a major effect of HuR on *Rgs4* splicing, we tested whether a different isoform is detected upon HuR knock-down by RT-PCR. We only detected the major annotated isoform *mmuRgs4*-201 (Sup. Fig. 1F). Together, this data suggests that *Rgs4* mRNA is rapidly degraded, resulting in a high turnover rate and that HuR is important for *Rgs4* destabilization in neurons. Therefore, we decided to investigate whether this relation is also reflected by the expression pattern of HuR protein and *Rgs4* mRNA.

### HuR protein and Rgs4 mRNA show divergent expression with neuronal maturation

We tested the expression pattern of HuR protein and *Rgs4* mRNA during neuronal maturation in cell culture. During maturation of neurons, neuronal processes grow out, build synaptic protrusions and finally connect to each other through fully functioning synapses [26]. Expression of the Hu proteins HuB/C/D has been well described in neurons [27,28]. The neuronal role of HuR, however, has only been recently investigated [16,17]. HuR protein expression decreased with neuronal maturation in our primary cortical neuron culture (Fig. 1D). In contrast to HuR protein, we found that *Rgs4* mRNA levels increased with neuronal maturation, measured both by qRT-PCR in hippocampal neurons (Fig. 1E) and by fluorescent *in situ* hybridization (FISH) against *Rgs4* in cortical neurons (Fig. 1F). Having established the relation between HuR and *Rgs4* mRNA in the endogenous context, we set out to define a possible HuR binding site in *Rgs4* mRNA.

### HuR represses Rgs4 expression through the Rgs4 3ʹ-UTR

**Figure 2.**
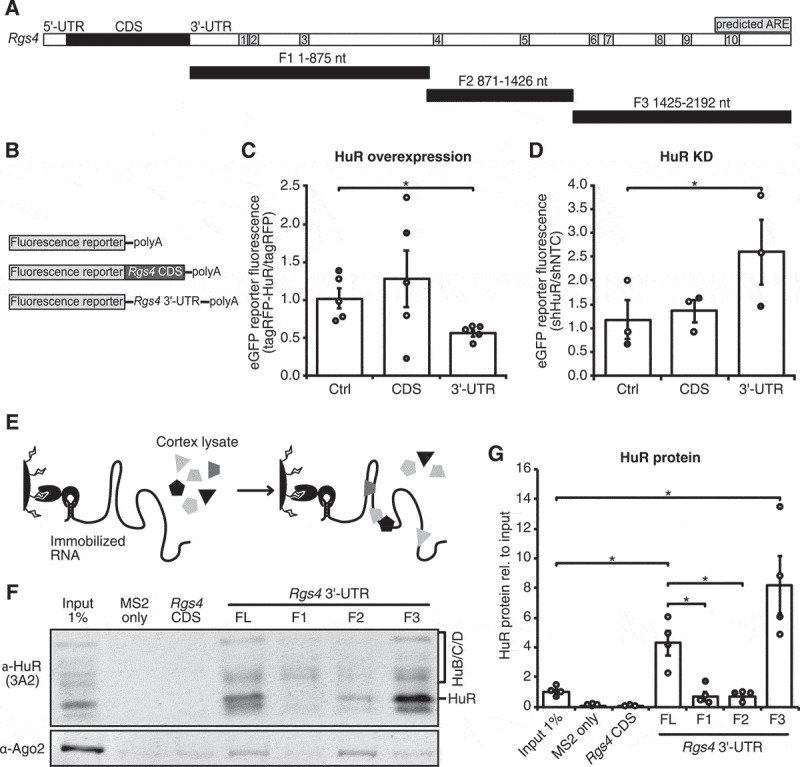
HuR represses *Rgs4* expression by binding to the 3ʹ end of *Rgs4* 3ʹ-UTR. (A) Scheme of *rnoRgs4* mRNA with predicted ARE sites and 3ʹ-UTR fragments used in (F,G). (B) Scheme of fluorescence reporter constructs used in (C,D). (C) Quantification of eGFP fluorescence intensity in the cell body of hippocampal neurons at 15 DIV co-transfected at 14 + 1 DIV with eGFP-reporter and tagRFP or tagRFP-HuR. Ratio of eGFP-reporter intensity between tagRFP-HuR and tagRFP condition is shown. Paired Student’s *t*-test. (D) Quantification of eGFP fluorescence intensity in the cell body of hippocampal neurons at 15 DIV transduced at 10 + 5 DIV with lentiviruses expressing shNTC or shHuR and transfected at 14 + 1 DIV with eGFP-reporter. Ratio of eGFP-reporter intensity between shHuR and shNTC condition is shown. Paired Student’s *t*-test. (E) Scheme of *in vitro* RNA affinity purification (TRAP) of RBPs based on immobilization of *in vitro* transcribed RNA via 2xMS2 stem loops. (F,G) Representative Western blot against HuR and Ago2 (F) and quantification (G) of HuR enrichment from adult rat cortex lysate in TRAP using 2xMS2 only, 2xMS2+ *Rgs4* CDS, 2xMS2+ *Rgs4* 3ʹ-UTR and different 2xMS2+ *Rgs4* 3ʹ-UTR fragments as depicted in (A) as bait RNA, normalized to input. Paired Student’s *t*-test. All error bars are SEM from ≥ 3 independent biological replicates; asterisks represent *p*-values (*p < 0.05). KD knock-down; NTC non-targeting control; DIV *days in vitro*; ARE AU-rich element

The coding sequence (CDS) of Rgs4 consists of 618 bases; the 3ʹ-UTR of 2,200 bases. We used the AREsite2 web database (http://rna.tbi.univie.ac.at/) to predict possible binding sites of HuR (Fig. 2A). Next, we used a fluorescent reporter assay consisting of eGFP only (Ctrl), a fusion protein of eGFP and *Rgs4* CDS and eGFP with the *Rgs4* 3ʹ-UTR to define whether HuR affects *Rgs4* CDS reporter or 3ʹ-UTR reporter expression (Fig. 2B). As shown in Fig. 2C, overexpression of tagRFP-HuR led to a decrease of *Rgs4* 3ʹ-UTR reporter, but not Ctrl or CDS reporter expression. Consistent with this, knock-down of HuR by shRNAs resulted in the opposite effect, an increase of *Rgs4* 3ʹ-UTR reporter, but not Ctrl or CDS reporter expression (Fig. 2D). Next, we defined the binding region of HuR in the *Rgs4* 3ʹ-UTR using either full-length (FL) or three different fragments of the *Rgs4* 3ʹ-UTR in an *in vitro* RNA purification experiment (trapping by RNA *in vitro* affinity purification; TRAP). For TRAP, the RNA of interest is tagged with two MS2 stem loops (2MS2) and transcribed *in vitro*. After immobilizing the RNA on amylose beads via a maltose binding and MS2 coat fusion protein (MBP-MCP), beads were incubated with lysate from adult rat cortices (Fig. 2E). Here, HuR protein was fourfold enriched when using the *Rgs4* 3ʹ-UTR FL RNA, but not by either CDS or MS2 only control RNA (Fig. 2F,G). Furthermore, the enrichment of HuR seems to be due to the binding of HuR to fragment 3, since fragment 1 and 2 did not show strong enrichment of HuR. Please note that binding of HuR was stronger in fragment 3 compared to *Rgs4* FL 3ʹ-UTR. This could be due to altered RNA folding or binding of additional RBPs. The RBP Ago2 yielded a different enrichment pattern in the TRAP assay with prominent enrichment in fragment 2 (Fig. 2F). Quantification of the signals of neuron-specific Hu proteins HuB/C/D, which all run slower than HuR, showed slight enrichment (1.4-fold) with the *Rgs4* FL 3ʹ-UTR as well as with fragment 1 and 3 (Sup. Fig. 2A,B). Together, we were able to show that binding of HuR takes place in the 3ʹ-end of the *Rgs4* 3ʹ-UTR; however, several AREs were predicted to be present in this fragment. Therefore, we decided to analyse the 3ʹ-UTR for additional predictable features, *e.g*. miRNA binding sites and sequence conservation.

### miR-26 represses Rgs4 by interacting with a conserved region

**Figure 3.**
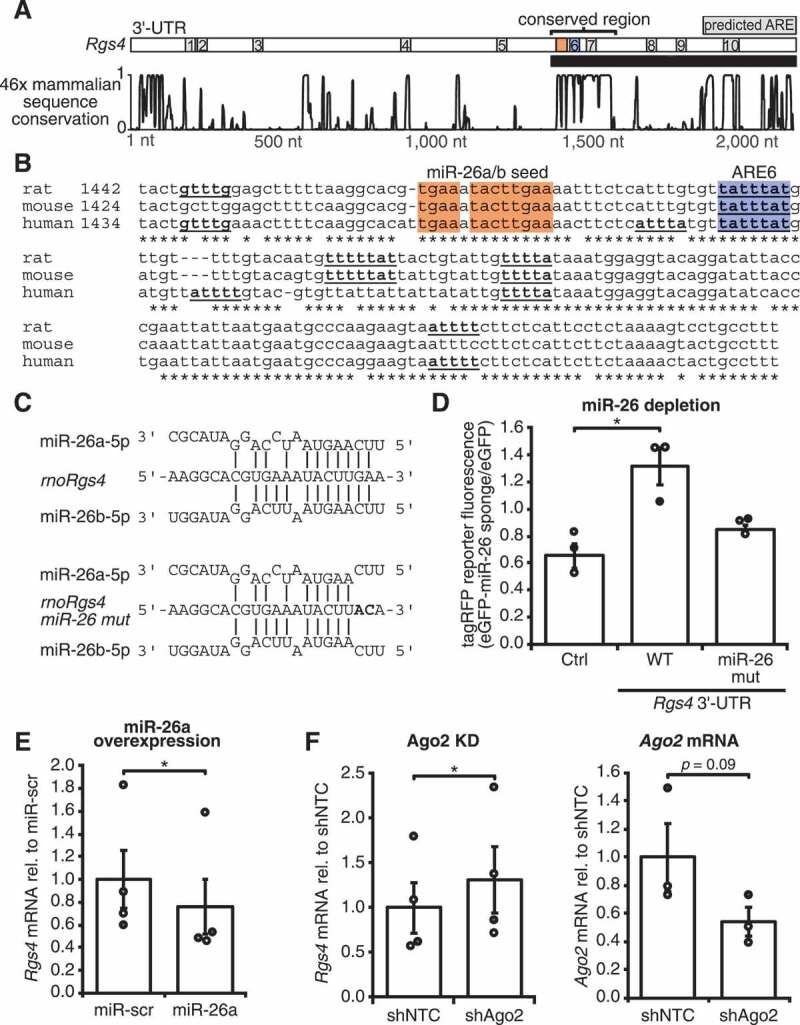
miR-26 represses *Rgs4* expression by binding to a conserved region. (A) Scheme of *Rgs4* 3ʹ-UTR with predicted AREs and miR-26 binding site (orange) and 46x mammalian sequence conservation of the *Rgs4* 3ʹ-UTR. (B) Comparison of rat, mouse and human RNA sequence of the conserved region depicted in (A). Predicted AREs are underlined, conserved ARE6 is highlighted in blue and miR-26a/b binding site is highlighted in orange. (C) Complementarity of rno-miR-26a and rno-miR-26b to WT *rnoRgs4* and miR-26 binding site mutant of *Rgs4* mRNA. (D) Quantification of tagRFP fluorescence intensity in the cell body of hippocampal neurons at 15 DIV transduced at 11 + 4 DIV with lentiviruses expressing eGFP or eGFP-miR-26 sponge (16x bulged miR-26a/b binding sites) and transfected at 14 + 1 DIV with tagRFP-reporter. Ratio of tagRFP-reporter intensity between eGFP-miR-26 sponge and eGFP condition is shown. Paired Student’s *t*-test. (E) Quantification of endogenous *Rgs4* mRNA by qRT-PCR in 14 DIV cortical neurons transduced at 11 + 3 DIV with lentiviruses expressing miR-scr or miR-26a, normalized to miR-scr. Paired Student’s *t*-test. (F) Quantification of endogenous *Rgs4* (left) and *Ago2* (right) mRNA by qRT-PCR in 14 DIV cortical neurons transduced at 9 + 5 DIV with lentiviruses expressing shNTC or shAgo2, normalized to shNTC. Paired Student’s *t*-test. All error bars are SEM from ≥ 3 independent biological replicates; asterisks represent *p*-values (*p < 0.05). KD knock-down; WT wild type; NTC non-targeting control; scr scrambled; ARE AU-rich element

In order to narrow down the HuR binding site(s), we analysed the sequence conservation in the *Rgs4* 3ʹ-UTR of over 46 different mammalian genomes. As shown in Fig. 3A, a highly conserved region in the third fragment was standing out, containing two conserved possible AREs, ARE6 (UAUUUAU) and 7 (UUUUA). Mainly positive effects of HuR on target expression levels have been previously reported [29]. In an interdependent mechanism together with the miRNA let-7, Gorospe et al. reported HuR to have a repressive effect on c-myc [22]. We asked whether the repressive effect of HuR on *Rgs4* mRNA was caused by other factors that associated with *Rgs4* mRNA. Using the prediction software TargetScanMouse7.2, we analysed *Rgs4* for predicted miRNA binding sites. Interestingly, the webserver found a conserved miR-26 8mer binding site within the conserved region of the *Rgs4* 3ʹ-UTR, in close proximity to the predicted ARE6 (Fig. 3B). miR-26 is a family of neuronal-enriched miRNAs [30,31], consisting of miR-26a and miR-26b, both shown to be important for neurogenesis [31], maintenance of long-term potentiation and dendritic spine enlargement [32]. As shown in Fig. 3C, miR-26a/b exhibit extended predicted binding to *Rgs4* mRNA in extension to the 8mer binding site, proposedly further strengthening the interaction. Introduction of two point mutations in the miR-26 binding site in *Rgs4* reduces complementarity and should abrogate miR-26 binding (Fig. 3C). We used a fluorescent reporter assay with tagRFP only (Ctrl), tagRFP fused to *Rgs4* 3ʹ-UTR, or the *Rgs4* 3ʹ-UTR miR-26 mutant to investigate the effect of miR-26 on Rgs4 expression. Coexpression of a miR-26a/b sponge construct fused to eGFP depleted the levels of free miR-26. As depicted in Fig. 3D, depletion of miR-26 led to an upregulation of the WT *Rgs4* 3ʹ-UTR reporter, but not the miR-26 mutant reporter, suggesting that miR-26 negatively regulates Rgs4 expression by binding to the conserved region. We next tested whether this effect could also be reproduced for endogenous Rgs4. Indeed, overexpression of miR-26a in cortical neurons led to a reduction of *Rgs4* mRNA levels, as measured by qRT-PCR (Fig. 3E). Furthermore, knock-down of Ago2, an essential RISC component, resulted in *Rgs4* mRNA upregulation (Fig. 3F). Overexpression of miR-26a or knock-down of Ago2 did not alter *HuR* mRNA levels (Sup. Fig. 3A,B). In sum, miR-26 is repressing *Rgs4* expression through interaction with a binding site within a conserved region of the *Rgs4* 3ʹ-UTR. This opens the question whether HuR and miR-26 synergistically act in reducing *Rgs4* mRNA levels.

### HuR and miR-26 show an interdependent mechanism of Rgs4 regulation

**Figure 4.**
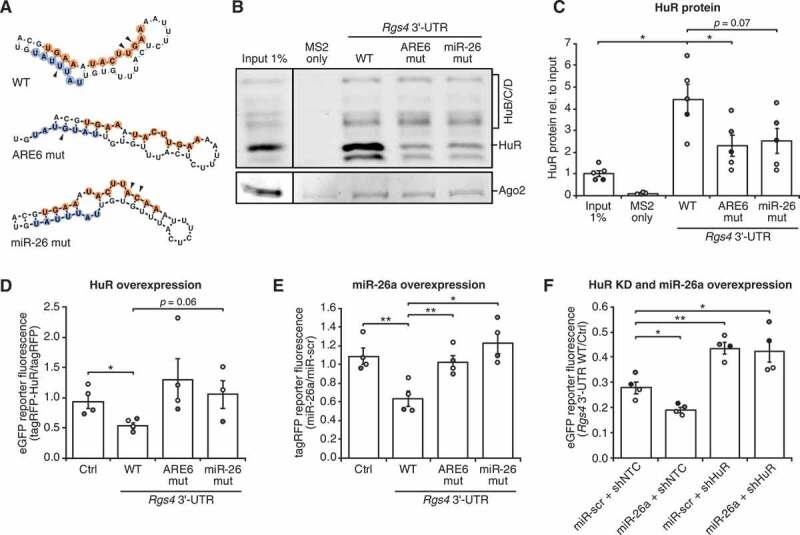
HuR and miR-26 synergistically repress *Rgs4* mRNA. (A) Predicted *in silico* folding of *Rgs4* conserved region (upper panel). The miR-26 binding site (orange) and the ARE6 (blue) are highlighted, with mutated sites marked by black arrows. (B,C) Representative Western blot against HuR and Ago2 (B) and quantification (C) of HuR enrichment from adult rat cortex lysate in *in vitro* RNA affinity purification using 2xMS2 only, 2xMS2+ *Rgs4* 3ʹ-UTR WT, 2xMS2+ *Rgs4* 3ʹ-UTR ARE6 mut and 2xMS2+ *Rgs4* 3ʹ-UTR miR-26 mut as bait RNA, normalized to input. Paired Student’s *t*-test. (D) Quantification of eGFP fluorescence intensity in the cell body of hippocampal neurons at 15 DIV co-transfected at 14 + 1 DIV with eGFP-reporter and tagRFP or tagRFP-HuR. Ratio of eGFP-reporter intensity between tagRFP-HuR and tagRFP condition is shown. Paired Student’s *t*-test. (E) Quantification of tagRFP fluorescence intensity in the cell body of hippocampal neurons at 15 DIV co-transfected at 14 + 1 DIV with tagRFP-reporter and miR-scr or miR-26a. Ratio of tagRFP-reporter intensity between miR-26a and miR-scr condition is shown. Paired Student’s *t*-test. (F) Quantification of eGFP fluorescence intensity in the cell body of hippocampal neurons at 15 DIV transduced at 10 + 5 DIV with lentiviruses expressing shNTC or shHuR and co-transfected at 14 + 1 DIV with eGFP-reporter and miR-scr or miR-26a. Ratio of eGFP-reporter intensity between *Rgs4* 3ʹUTR WT and Ctrl reporter is shown. Paired Student’s *t*-test. All error bars are SEM from ≥ 3 independent biological replicates; asterisks represent *p*-values (*p < 0.05, **p < 0.01). ARE AU-rich element; WT wild type; KD knock-down; Scr scrambled

To explore the working model that HuR destabilized *Rgs4* mRNA by an interdependent effect with miR-26, we examined the conserved region for predicted secondary structures. Using thermodynamic structure prediction (RNAfold), we found the HuR and the miR-26 binding site to be in close proximity within the same RNA hairpin structure (Fig. 4A, Sup. Fig. 4A). We hypothesize a model where both miR-26 and HuR association with the RNA are needed to open the hairpin structure and enable sufficient repression of *Rgs4*. Using the TRAP assay, we evaluated the binding of HuR to WT, ARE6 mutant or miR-26 mutant *Rgs4* 3ʹ-UTR RNAs. Neither of the mutants led to major changes of the predicted hairpin structures or significantly changed the minimal free energy of miR-26 binding to *Rgs4* mRNA (Fig. 4A, Sup. Fig. 4A,B). As shown in Fig. 4B, both mutating the HuR binding site (ARE6 mut) and the miR-26 binding site (miR-26 mut) significantly reduced HuR association with *Rgs4* 3ʹ-UTR supporting our hypothesis of an interdependent mechanism. Binding of HuB/C/D to *Rgs4* 3ʹ-UTR was unaffected by the mutations (Sup. Fig. 4C). Finally, we aimed at validating our results from the *in vitro* binding assay in hippocampal neurons using fluorescent reporter assays. Fig. 4D shows that overexpression of tagRFP-HuR led to a significant reduction of WT *Rgs4* 3ʹ-UTR eGFP-reporter expression, but not of *Rgs4* 3ʹ-UTR eGFP-reporter containing either HuR or miR-26 binding site mutants. Complementing this experiment, we used the tagRFP-reporter assay to test for the effect of miR-26a overexpression on WT, ARE6 and miR-26 *Rgs4* 3ʹ-UTR tagRFP-reporter expression. Overexpression of miR-26a resulted in significant downregulation of WT *Rgs4* 3ʹ-UTR tagRFP-reporter expression, but not of the *Rgs4* 3ʹ-UTR tagRFP-reporter containing the HuR or miR-26 binding site mutants (Fig. 4E).

### HuR is necessary for the repressive effect of miR-26a on Rgs4 mRNA

To substantiate the observed interdependent mechanism from the mutation studies, we tested whether miR-26a represses *Rgs4* 3ʹ-UTR reporter, when HuR protein has been depleted. While sole overexpression of miR-26a led to reduction of eGFP-*Rgs4* 3ʹ-UTR reporter expression, the effect was abolished when HuR levels were depleted (Fig. 4F). Furthermore, we investigated, whether the effects of HuR and miR-26a are additive using the eGFP reporter assay. In this case overexpression of both HuR and miR-26a would result in stronger repression of *Rgs4* 3ʹ-UTR reporter expression, compared to overexpression of either HuR or miR-26a. However, we did not detect further repression, when both HuR and miR-26a were overexpressed (Sup. Fig. 4D). Together, our results from the mutation and the HuR/miR-26a combination studies substantiate the model of an interdependent mechanism of HuR and miR-26 in repressing *Rgs4* mRNA expression.

### Mutation of miR-26 and HuR binding sites increases dendritic Rgs4 mRNA levels

**Figure 5.**
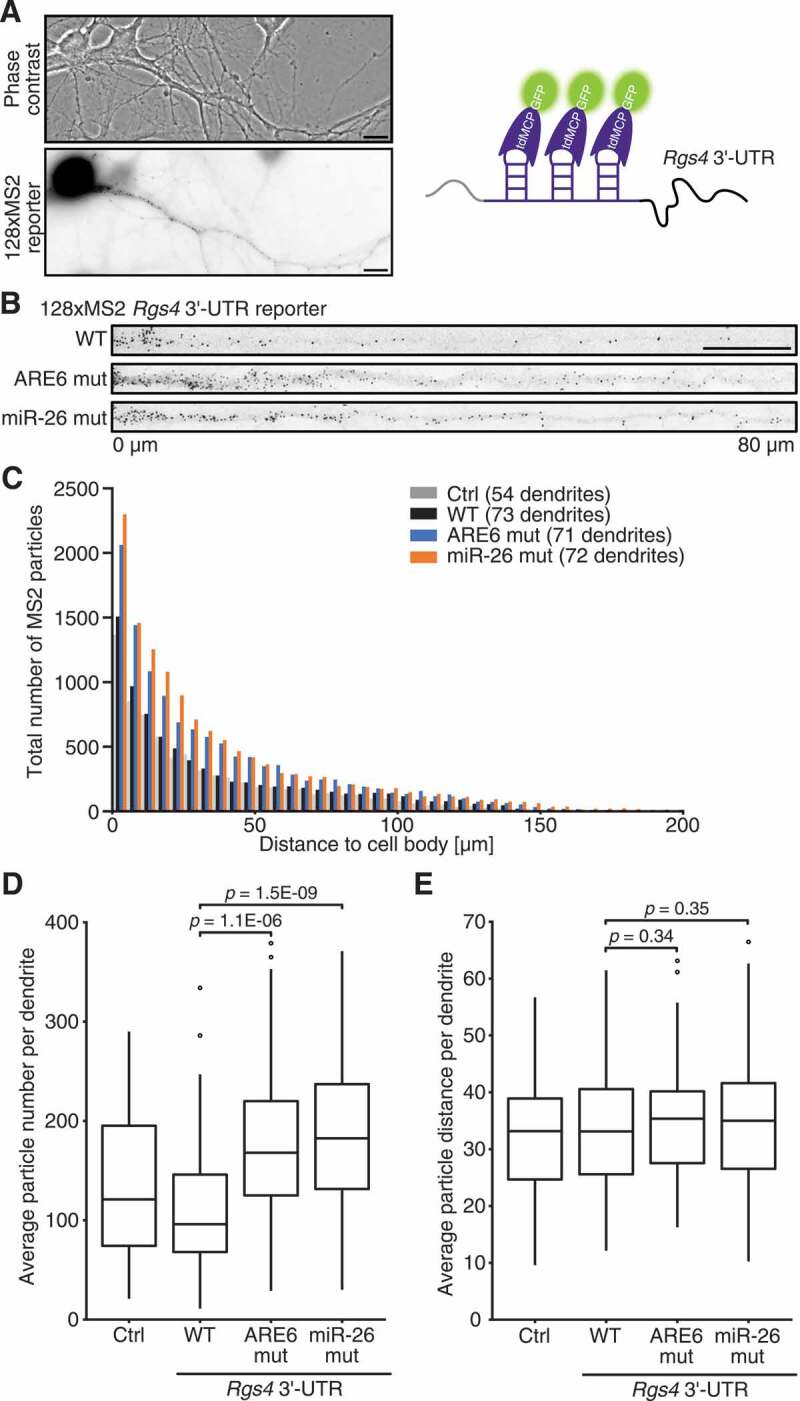
Mutation of miR-26 and HuR binding sites increases dendritic *Rgs4* RNA levels. (A) Phase contrast and 128xMS2 GFP reporter fluorescence in a rat hippocampal neuron at 14 + 1 DIV expressing both tdMCP-GFP and 128xMS2+ *Rgs4* 3ʹ-UTR reporter RNA (left panel). Scheme of tdMCP-GFP bound to MS2+ *Rgs4* reporter RNA (right panel). (B) Deconvolved and straightened images of dendrites expressing both tdMCP-GFP and 128xMS2+ *Rgs4* 3ʹ-UTR WT, ARE6 mut or miR-26 mut reporter mRNA. Straightened images are cropped to 80 µm for better particle visibility. (C) Histogram displaying MS2 particle distance to cell body and total number of MS2 particles from hippocampal neurons transfected with tdMCP-GFP and 128xMS2+ Ctrl, 128xMS2+ *Rgs4* 3ʹ-UTR WT, ARE6 mut or miR-26 mut reporter mRNA at 14 + 1 DIV. Binning on x-axis is 5 µm. (D) Boxplot of the average number of MS2 particles per dendrite. Unpaired Student’s *t*-test. (E) Boxplot of the average distance of MS2 particles per dendrite. Unpaired Student’s *t*-test. Data are obtained from 3 independent biological replicates; Dendrites: Ctrl n = 54, WT n = 73, ARE6 mut n = 71, miR-26 mut n = 72; scale bar 10 µm. ARE AU-rich element; WT wild type; tdMCP-GFP tandem MS2 coat protein fused to GFP; DIV *days in vitro.*

We finally aimed to investigate whether miR-26 and HuR could affect dendritic *Rgs4* mRNA levels. For this, we used the MS2 reporter system, previously applied to study live dynamics of *Rgs4* 3ʹ-UTR [14]. The system is based on a reporter containing a LacZ open reading frame and a repetition of 128xMS2 stem loops in front of a 3ʹ-UTR of interest. Hippocampal neurons were co-transfected with the 128xMS2 reporter and tandem MS2 coat protein-GFP (tdMCP-GFP) (Fig. 5A). Binding of the tdMCP-GFP to the MS2 stem loops in the 128xMS2 reporter visualized the RNA reporter and allowed the quantification of dendritic MS2 particles (Fig. 5B). We measured the distance of dendritic MS2 particles from the cell body and counted the total number of dendritic MS2 particles for 128xMS2 reporter with either no 3ʹ-UTR (Ctrl), WT, ARE6 mutant and miR-26 mutant *Rgs4* 3ʹ-UTR (Fig. 5C). As visualized in Fig. 5D, the average particle number per dendrite was significantly higher for the *Rgs4* 3ʹ-UTR mutant reporters compared to WT. However, the distribution of MS2 particles along the dendrite was not affected by mutating either the ARE6 or the miR-26 binding site (Fig. 5E, Sup. Fig. 5A). This data on *Rsg4* 3ʹ-UTR RNA reporter expression and localization suggests that HuR and miR-26 destabilize dendritic *Rgs4* mRNA, while not directly affecting *Rgs4* localization. This was important, as HuR is predominantly located in the nucleus under basal conditions, with scarce localization to dendrites [33,34].

In conclusion, our data suggest that miR-26/RISC and HuR co-regulate *Rgs4* 3ʹ-UTR, resulting in destabilization of the mRNA. This is consistent with our working model of a dynamic RNA conformation, where binding of miR-26/RISC and HuR acts as a switch in opening an RNA hairpin structure. This enables a strong repression of *Rgs4* mRNA by downstream effectors. In mature neurons, however, the absence of HuR together with the binding of additional RBPs could favour the hairpin structure (‘closed conformation’), thereby preventing miR-26/RISC binding. This, in turn, results in increased mRNA levels of *Rgs4*, coding for a protein that plays a critical role in the regulation of synaptic plasticity.

## Discussion

**Figure 6.**
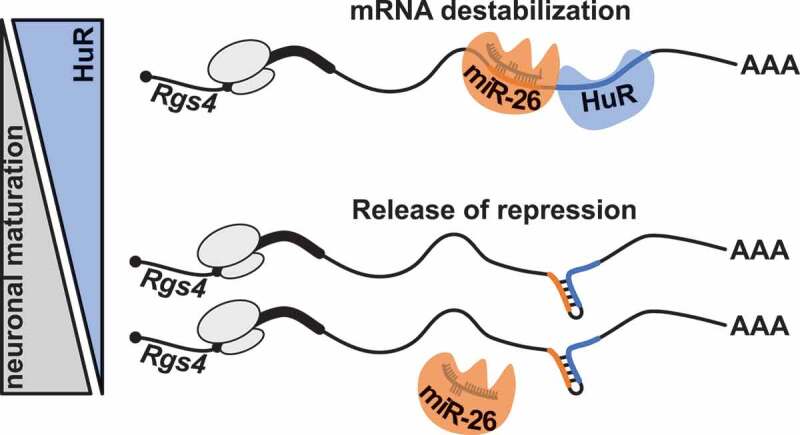
Proposed model of synergistic action of HuR and miR-26/RISC in neurons. Both, miR-26/RISC and HuR, are needed to open up the hairpin structure in *Rgs4* 3ʹ-UTR and enable stable binding of both factors. This results in destabilization of *Rgs4* mRNA. The decrease of HuR protein with neuronal maturation or binding of additional RBPs to the mRNA favours the hairpin structure and miR-26/RISC can no longer bind. This results in increased mRNA levels of *Rgs4*, coding for a protein important for regulation of neuronal activity. See text for further details

In our study, we provide strong experimental evidence that miR-26 and HuR destabilize *Rgs4* mRNA in a synergistic manner. In contrast to previous studies in smooth muscle cells, where *Rgs4* mRNA was stabilized by HuR [7] and Rgs4 overexpression rescued the phenotype observed in HuR knock-down cells [21], we find the opposite effect of HuR on *Rgs4* mRNA in neurons (Fig. 1). Furthermore, we identified the ARE bound by HuR to be within a conserved region in the 3ʹ end of the *Rgs4* 3ʹ-UTR (Figs. 2 and 4). Analysis of the conserved region revealed a functional miR-26a/b binding site in close proximity to the ARE (Fig. 3). So far, HuR mainly exerts a stabilizing effect on target mRNAs, often by competing with miRNA binding [23,25]. There is, however, also evidence that HuR can destabilize mRNAs by cohesive action with miRNAs [24]. Based on the data presented in Fig. 4, we conclude that there is indeed evidence for synergism of miR-26 and HuR in repressing *Rgs4* mRNA. Mutation of both the miR-26 binding site and the ARE results in reduced binding of HuR in the TRAP assay. Further, we show that the miR-26 binding site mutant abolishes HuR and the ARE mutant abolishes miR-26 repressive effect on the *Rgs4* 3ʹ-UTR reporter expression. Finally, in an experiment independent of mutations in the 3ʹ-UTR sequence, we show that HuR is necessary for miR-26 to repress the *Rgs4* 3ʹ-UTR reporter (Fig. 4F). Our data support a model, where both miR-26 and HuR can bind to the *Rgs4* 3ʹ-UTR in order to facilitate repression of *Rgs4* mRNA. We, therefore, hypothesize that reduced HuR levels in mature neurons (Fig. 1) lead to deregulation of *Rgs4* mRNA by both miR-26 and HuR. This would result in increased Rgs4 expression, important for proper neuronal function [5]. However, we cannot (yet) reliably define an order of events. In our presented model, the synergistic repressive effect of HuR and miR-26 on *Rgs4* mRNA arises from binding to an RNA sequence predicted to form a hairpin structure (Fig. 6). This assumption is based on RNA folding prediction, as we cannot yet provide experimental support for the predicted secondary RNA structure. Extensive future work is therefore needed to experimentally validate the change in RNA structure. An elegant study by Kim *et al*. (2009) illustrated a similar interaction mode of HuR and let-7 loaded Ago/RISC on *c-myc* mRNA [22].

Further support of our model comes from a genome-wide study by Li *et al*. (2018), which recently showed both antagonistic and agonistic interaction modes between HuR and the miRNA machinery [24]. They used high throughput sequencing of RNA after crosslinking and immunoprecipitation (CLIP) to map HuR or Ago2 binding sites and studied the effect of HuR knock-down on mRNA occupancy of Ago2 in human embryonic kidney cells. In addition to future studies addressing the combinatorial function of different RBPs as well as the miRNA/RISC machinery in brain through CLIP, it will be intriguing to get insight into the RBP-dependent dynamics of RNA structures in living cells [35]. To our current knowledge, the regulation of the AU-rich transcriptome in the brain has been studied solely through the neuron-specific Hu proteins HuB/C/D [36–39]. Only recently, the neuronal function of the ubiquitously expressed HuR has been explored [17–19,40,41]. It will, therefore, be interesting to see in the future, whether the data on post-transcriptional regulation by HuR from non-neuronal cells and tissues hold true for the complex nervous system. As our data show, there is no uniform mechanism of mRNA regulation by HuR, highlighting the importance of mechanistic studies on the single target level.

## Materials and methods

### Plasmids

The expression plasmid for MBP-MS2BP (Addgene 11,246) [42,43] and the pUBC-NLS-ha-tdMCP-GFP [14] and pRSV-LacZ-128xMS2 [14] plasmids have been described. Generation of pRSV-LacZ-128xMS2, pCMV-tagRFP-STOP (ptagRFP-C, Evrogen) or pCMV-eGFP-STOP [44] (pEGFP-C1, Clontech) reporter plasmids was performed by insertion of the CDS (position 110–727 nt) or 3ʹ-UTR (position 728–2919 nt) of rat *Rgs4* mRNA (NM_017214.1) in between the stop codon of the respective open reading frame of Lacz, tagRFP or eGFP and the poly A signal. Fragments of the Rgs4 3ʹ-UTR were cloned by insertion of fragments of rat *Rgs4* mRNA (NM_017214.1), corresponding to position 728–1602 nt (F1), 1698–2153 nt (F2), 2152–2919 nt (F3), into pCMV-eGFP-STOP reporter as described above. Site-directed mutagenesis was performed on pRSV-LacZ-128xMS2-*Rgs4* 3ʹ-UTR, pCMV-tagRFP-*Rgs4* 3ʹ-UTR and pCMV-eGFP-*Rgs4* 3ʹ-UTR reporter plasmids to generate *Rgs4* 3ʹ-UTR ARE6 and *Rgs4* 3ʹ-UTR miR-26 mutant reporter plasmids using the following primers (5′–3′): Rgs4_ARE6_F: ctcatttgtgttatGtatgttgttttg; Rgs4_ARE6_R: caaaacaacataCataacacaa-atgag; Rgs4_miR-26_F: cacgtgaaatacttACaaatttctc; Rgs4_miR-26_R: gagaaatttGTa-agtatttcacgtg. The *in vitro* RNA transcription plasmid pcDNA3-T7-Ctrl-2xMS2 has been previously described [43]. The plasmids pcDNA3-T7-2xMS2 containing *Rgs4* CDS, 3ʹ-UTR, 3ʹ-UTR fragments and mutants were generated by subcloning the insert from respective above mentioned pCMV-eGFP-STOP reporter plasmids in between the T7-promotor and the 2xMS2 sequence by EcoRV/BamHI. pCMV-tagRFP-HuR was generated by inserting the coding sequence of HuR, corresponding to position 153–1133 nt of rat *HuR* mRNA (NM_001108848), into ptagRFP-C (evrogen). The plasmid pSup-eGFP-H1-pri-miR-26a used in the tagRFP-assay was generated by PCR-amplification and cloning of the primary rno-miR-26a sequence after the H1 promotor into the pSuperior.neo+GFP (oligo engine) using primer annealing 60 nt up- and downstream of the genomic locus of the rno-miR-26a stem-loop. Generation of pSup-eGFP-H1-miR-scr was performed by annealing and direct ligation of the following phosphorylated oligos after the H1 promotor into pSuperior.neo+GFP: miR-scr_F: gatccccgtgtaacacgtctatacgcccattcaagagatgggcgtatagacgtgttacacttttta; miR-scr_R: ag-cttaaaaagtgtaacacgtctatacgcccatctcttgaatgggcgtatagacgtgttacacggg. For combining HuR and miR-26a overexpression in eGFP-reporter assays, the following expression plasmids were generated: pSup-tagBFP-H1-miR-scr and pSup-tagBFP-H1-pri-miR-26a, by exchanging the eGFP open reading frame by tagBFP in the above-described pSup-eGFP plasmids. For generation of the lentiviral plasmids pFu3a-H1-pri-miR-26a-pCamK2a-tagRFP and pFu3a-H1-miR-scr-pCamK2a-tagRFP, the H1-shNTC sequence of the previously described pFu3a-H1-shNTC-pCamK2a-tagRFP [14] was exchanged by the H1-pri-miR-26a or H1-miR-scr sequence from above described pSup-eGFP-H1. Generation of the lentiviral plasmids pFu3a-pCamK2a-eGFP-STOP and pFu3a-pCamK2a-eGFP-STOP-16xmiR-26sponge was performed by first exchanging the H1-shNTC-pCamK2a-tagRFP by pCamK2a-eGFP-STOP. For pFu3a-pCamK2a-eGFP-STOP-16xmiR-26sponge, the following phosphorylated oligos were annealed, ligated and separated by 2% agarose gel electrophoresis (5′–3′): miR26a_sponge_F: ccggcagcctatcctCCttacttgaac; miR26a_sponge_R: ccgggttcaagtaaGGaggataggctg; 5ʹlinker_sponge_F: agat-ctcgagctcaagcttcgaattcc; 5ʹlinker_sponge_R: ccggggaattcgaagcttgagctcgagatct, 3ʹlinker_sponge_F: ccggcgtcgacggtaccgcgggcccgggatcc; 3ʹlinker_sponge_R: ggatcc-cgggcccgcggtaccgtcgacg. A band of ~500 bp was excised, gel purified and pasted into the 3ʹ-UTR of eGFP into pFu3a-pCamK2a-eGFP-STOP via BamH/XhoI. To generate the lentiviral shRNA plasmids pFu3a-H1-shHuR-pCamK2a-tagRFP, pFu3a-H1-shAgo2-pCamK2a-tagRFP and pFu3a-H1-shPum2-pCamK2a-tagRFP, the H1-shNTC sequence of pFu3a-H1-shNTC-pCamK2a-tagRFP was exchanged by H1-shHuR, H1-shAgo2 or H1-shPum2 after subcloning the shRNAs into pSuperior.neo+GFP. The oligo sequences for shRNA generation were (5′–3′): shHuR_F: gatccccgaagaggcaatta-ccagtttcattcaagagatgaaactggtaattgcctcttctttttc; shHuR_R: tcgagaaaaagaagaggcaatta-ccagtttcatctcttgaatgaaactggtaattgcctcttcggg; shAgo2_F: gatcccctgttcgtgaatttgggatcat-tgtacaatgatcccaaattcacgaacatttttc; shAgo2_R: tcgagaaaaatgttcgtgaatttgggatcattgtac-aatgatcccaaattcacgaacaggg; shPum2_F: gatccccaccaagttggtctggattcttcaagagagaatc-cagaccaacttggttttttc; shPum2_R: gaaaaaaccaagttggtctggattctctcttgaagaatccagaccaa-cttggtggggatc. The lentiviral packaging plasmids, psPAX2 and pcDNA3.1-VSV-G, have previously been described [12].

### Lentivirus production

Lentiviral particles for shNTC, shHuR, shAgo2, miR-scr, miR-26a, eGFP-Stop and eGFP-miR-26-sponge were generated from HEK-293 T cells co-transfected with psPAX2, pcDNA3.1-VSV-G and the respective pFu3a plasmids using calcium phosphate coprecipitation. After 48 h virus production, supernatants were filtered (0.45 µm PVDF Millex-HV; Millipore), concentrated by ultracentrifugation (65,000xg, 140 min, SW 32 Ti rotor; Beckman Coulter) and resuspended in Opti-MEM™ (Life Technologies) [12].

### Neuronal cell culture, treatment, transduction and transfection

All animals in this study were used according to the German Welfare for Experimental Animals (LMU Munich, *Regierung von Oberbayern*). Rat hippocampal neuron cell cultures from embryos at day 17 (E17) of timed pregnant Sprague-Dawley rats (Charles River Laboratories) were generated as described previously [45]. Briefly, E17 hippocampi were dissected, trypsinized and cells dissociated and plated on poly-L-lysine-coated coverslips and cultured in NMEM+B27 medium (Invitrogen) with 5% CO_2_ at 37°C. For cortical cultures, E17 cortices were trypsinized and dissociated, the cell suspension sequentially filtered through 100-, 70- and 40-μm cell strainers and then plated at a density of 100,000 cells/cm^2^ on poly-L-lysine coated 60 mm dishes. For protein and RNA analysis, cortical neurons were transduced with lentiviral suspension at 9 days *in vitro* (DIV) and lysed at 14 DIV. Analysis of RNA stability was performed by incubation of lentivirus-treated cortical neurons at 14 DIV with 2 µM Actinomycin D (ActD; Sigma) or an equivalent amount of DMSO in NMEM+B27 for 90 min. Hippocampal neurons were transduced with lentiviral suspension at 10–11 DIV, followed by transient transfection by calcium phosphate coprecipitation [46] at 14 DIV and fixation with 4% paraformaldehyde (PFA) at 15 DIV. Transient co-transfection of hippocampal neurons by calcium phosphate precipitation was performed at 14 DIV, followed by fixation with 4% PFA at 15 DIV.

### Fluorescent reporter assays

For fluorescent reporter assays with mere overexpression of miR-26a, HuR or both, hippocampal neurons grown on coverslips were transiently co-transfected at 14 DIV with the respective overexpression plasmids and the fluorescent reporter constructs. Neurons were fixed with 4% PFA 24 h post-transfection at 15 DIV. For fluorescent reporter assays with knock-down of HuR or depletion of miR-26, hippocampal neurons grown on coverslips were transduced with lentiviruses at 10 DIV for HuR knock-down and at 11 DIV for miR-26a depletion. At 14 DIV, neurons were transfected with the fluorescent reporter constructs (and miR-26a overexpression constructs to combine shHuR and miR-26a overexpression) followed by fixation with 4% PFA 24 h post-transfection at 15 DIV. Coverslips were mounted on microscope slides with Fluoromount™ Aqueous Mounting Medium (Sigma), imaged and analysed as described in the microscopy and image analysis section.

### Protein purification

The MBP-MCP fusion protein was affinity purified as described [42] using amylose resin (New England Biolabs) in MBP-buffer (20 mM Tris at pH 7.2, 50 mM NaCl; 1 mM EDTA) and step elution with 10 mM maltose. Further purification was performed by linear NaCl elution from a heparin column using an Äkta purifier (GE Healthcare). Eluted fractions were combined, concentrated and washed with binding buffer (BB: 20 mM Tris, pH 7.5, 150 mM NaCl, 1.5 mM MgCl_2_, 8.7% glycerol and 0.05% NP40) using Amicon Ultra centrifugal filters (Merck).

### *In vitro* RNA affinity purification

*In vitro* RNA affinity purification was performed as previously described [43] with minor variations. Briefly, RNA containing 2xSM2 stem loops was *in vitro* transcribed by run-off transcription from linearized (XhoI) pcDNA3.1-T7-MS2 plasmids using the T7 RiboMAX Express Large-Scale RNA Production System (Promega). Synthesized RNAs were purified using NucAway spin columns (Invitrogen). Twenty microlitres of amylose resin (New England Biolabs) was washed four times with BB and incubated with 100 pmol recombinant MBP-MCP in 1 ml BB for 30 min. The resin was blocked with 0.5 mg/ml bovine serum albumin in 1 ml BB for 30 min and washed three times with binding buffer (BB). Twenty picomoles i*n vitro* transcribed bait RNA was heated to 65°C for 10 min, let cool to room temperature over 10 min and immobilized on the resin 1 ml BB + 11 mg/mL heparin (Sigma) for 1 h. One adult rat cortex was lysed in 1 ml BB + cOmplete Protease Inhibitor Cocktail Tablets (Roche) using bead homogenization on a FastPrep-24 instrument (MP Biomedicals) with lysing matrix D (MP Biomedicals). The lysate was diluted to 1:20 with BB + cOmplete Protease Inhibitor and cleared twice by centrifugation at 15.600xg and 4°C for 10 min. The RNA loaded resin was washed once with BB + 11 mg/ml heparin, before the resin was incubated for 30 min with 500 µl lysate and 500 µl BB + 22 mg/ml heparin, 2 mM dithiothreitol and 40 U/ml murine RNase Inhibitor (New England Biolabs). The resin was washed four times with BB + 11 mg/ml heparin. Proteins were eluted by incubation with 15 µl 3x SDS sample buffer at 65°C for 12 min. All steps, except lysis, RNA folding and elution, were conducted at room temperature and constant agitation.

### Western blotting

Neurons were washed twice with warm Hanks′ Balanced Salt Solution (HBSS, Gibco) and then lysed in 3x SDS sample buffer. Samples were treated with 50 U Benzonase Nuclease (Merck) for 10 min and heated to 65°C for 12 min. Proteins of equivalent number of neurons were resolved on 10% SDS-PAGE and subjected to immunoblotting with mouse anti-HuR (3A2) (1:500, sc-5261, Santa Cruz), mouse anti-Ago2 (2E12-1 C9) (1:500, WH0027161M1, Sigma) goat anti-Vinculin (1:500, sc-7649, Santa Cruz). After incubation with IRDye labelled secondary donkey anti-mouse (IRDye 800CW) and donkey anti-goat (IRDye 680RD) (both 1:15,000, Li-Cor), membranes were imaged on an Odyssey CLx Imaging System (Li-Cor) and quantified using Image Studio Lite software (Li-Cor).

### RNA extraction, cDNA synthesis and qRT-PCR

Neurons were washed twice with warm HBSS (Gibco), before total RNA from cortical neurons was extracted using TRIzol (Invitrogen) and total RNA from hippocampal neurons grown on coverslips was extracted using RNeasy Mini Kit (Qiagen). cDNA was generated from 1 µg of DNase treated total RNA, using Superscript III reverse transcriptase (Invitrogen) according to the manufacturer’s instructions with a minor variation. A mixture of 1.5 µM random primer mix (New England Biolabs) and 2.5 μM (dT)20 was used during cDNA synthesis. Quantitative real-time PCR (qRT-PCR) was performed in duplicates from a 1:50 dilution of the stock cDNA using a home-made SYBR Green Master Mix [44], with the LightCycler 96 System (Roche). Only primers with an optimized efficiency of 95–105% were used. The 2^−ΔΔCt^ method implemented in the LightCycler Software (Roche) was used to calculate differences in RNA levels relative to peptidylprolyl isomerase A (PPIA) mRNA. The sequences of the qRT-PCR primers were (5′–3′): Ppia_F: gtcaaccccaccgtgttctt; Ppia_R: ctgctgtctttggaactttg; Rgs4_F: agtcccaaggccaagaagat; Rgs4_R: aacatgttccggcttgtctc; HuR_F: tcggtttgggcgaatcatca; HuR_R: ctagcaggcgagtggtacag; Ago2_F: acaagctggttttgcgctac; Ago2_R: ttgctgatctcct-cttgccg; Pum2_F: atgggagcagctctttgact; Pum2_R: gatgagccaaatccactgagag. Reverse transcription PCR (RT-PCR) was performed from a 1:50 dilution of the cDNA using *Taq* DNA Polymerase (NEB) according to the manufacturer instructions. The sequences of the RT-PCR primers were (5′–3′): Rgs4_RT_F: aatagaaaccaccgttgctc; Rgs4_RT_R: aacatgttccggcttgtctc.

### FISH and immunostaining

For FISH and immunostaining neurons were washed twice with warm HBSS and then fixed with warm 4% PFA in HBSS for 10 min. The fluorescence *in situ* hybridization (FISH) against *Rgs4* mRNA using Cy5-tyramide signal amplification was performed as described [12,47]. For immunostaining, fixed cells were washed with HBSS and permeabilized with 0.1% Triton X-100 in DPBS for 5 min. The following primary antibodies were used: mouse anti-HuR (3A2) (1:500, sc-5261, Santa Cruz Biotechnology) and mouse anti-Map2 (HM-2) (1:500, M4403, Sigma). The following secondary antibodies were used: donkey anti-mouse AF488- or AF647-conjugated antibodies (both Invitrogen). Coverslips were mounted on microscope slides with Prolong Diamond antifade mounting medium (Invitrogen).

### Microscopy and image analysis

Images were acquired using Zeiss Zen software on a Zeiss Z1 Axio Observer microscope including a 63x Plan-Apochromat oil immersion objective (1.40 NA), a COLIBRI.2 LED and an HXP 120 C light source and the Axiocam 506 mono camera. Neurons were selected for cell morphology and viability as well as for expression of plasmids and images were taken of the dendritic plane. For FISH experiments, z-stacks of neurons were acquired (50 images with 0.26 µm step-size), and a z-projection of the maximum intensity was performed in ImageJ. For cell body, fluorescence intensity quantification of eGFP- or tagRFP-reporter signal, the measure function in the Zeiss Zen software was used and a region of interest was drawn by hand based on the phase-contrast image. The mean intensity of each condition was calculated and normalized to the fluorescent reporter levels in the control conditions (miR-scr or tagRFP) with one exception. For experiments with overexpression or knock-down of both miR-26a and HuR, the mean intensity of the reporter fluorescence was normalized to the control eGFP reporter. For 128xMS2 experiments, z-stacks of neurons were acquired (30 images with 0.26 µm step-size). Images were then deconvoluted using the Zeiss Zen software deconvolution module, with default settings of the constrained iterative method and analysed in ImageJ. A z-projection of the maximum intensity was performed in ImageJ, and for 128xMS2 particle quantification, one dendrite per cell was selected and straightened using the segmented line tool with 40-pixel width. Particles were manually detected using the multipoint tool and the ROI manager. The distance was measured by extracting the x position for each particle in µm. The average number and average distance of particles per dendrite were calculated. For all experiments, ≥20 dendrites or cell bodies per condition from at least three independent experiments were selected for quantification.

### RNA structure and binding site predictions

The thermodynamic structure prediction of the conserved region corresponding to nucleotide position 1442 to 1631 of the 3ʹ-UTR sequence of rat *Rgs4* mRNA (NM_017214.1) was predicted using the RNAfold server within the ViennaRNA web services (http://rna.tbi.univie.ac.at/) [48]. Standard options were used, but no GU pairs at the end of helices were allowed. Accessibility of the miRNA interaction site within *Rgs4* 3ʹ-UTR conserved region was predicted with IntaRNA web interface within the Freiburg RNA tools (http://rna.informatik.uni-freiburg.de/IntaRNA) [49]. Prediction of ARE in the mouse *Rgs4* 3ʹ-UTR was performed using AREsite2 within the ViennaRNA web services (http://rna.tbi.univie.ac.at/) [50].

### Statistical analysis

Microsoft Excel and R software were used for data processing, plotting and statistical analysis [51,52]. Figures represent mean ± standard error of the mean (SEM) of at least three independent biological replicates. Asterisks represent p-values obtained by one-way ANOVA and either paired or unpaired two-sided Student’s t-test using the mean values per experiment (*p < 0.05, **p < 0.01, ***p < 0.001), as indicated.

## Supplementary Material

Supplemental Material
