# Supplementary material for: Synergistic regulation of Rgs4 mRNA by HuR and miR-26/RISC in neurons
Source: RNA Biol. 2020 Aug 11;18(7):988–98. doi: 10.1080/15476286.2020.1795409 (PMC8216180; doi:10.1080/15476286.2020.1795409)
Supplement: Supplemental Material [file KRNB_A_1795409_SM2160.pdf]

Supplementary Information

Synergistic regulation of *Rgs4* mRNA by HuR and miR-26/RISC in neurons

Authors

Janina Ehses [a], Sandra M. Fernández-Moya [a], Luise Schröger [a], Michael A. Kiebler [a]

[a] BioMedical Center, Medical Faculty, Ludwig Maximilians University of Munich, Martinsried, Germany

Corresponding Author:

Michael A. Kiebler (mkiebler@lmu.de)

Department of Cell Biology, BioMedical Center, Ludwig Maximilians University of Munich, Großhaderner Str. 9, 82152 Planegg-Martinsried, Germany

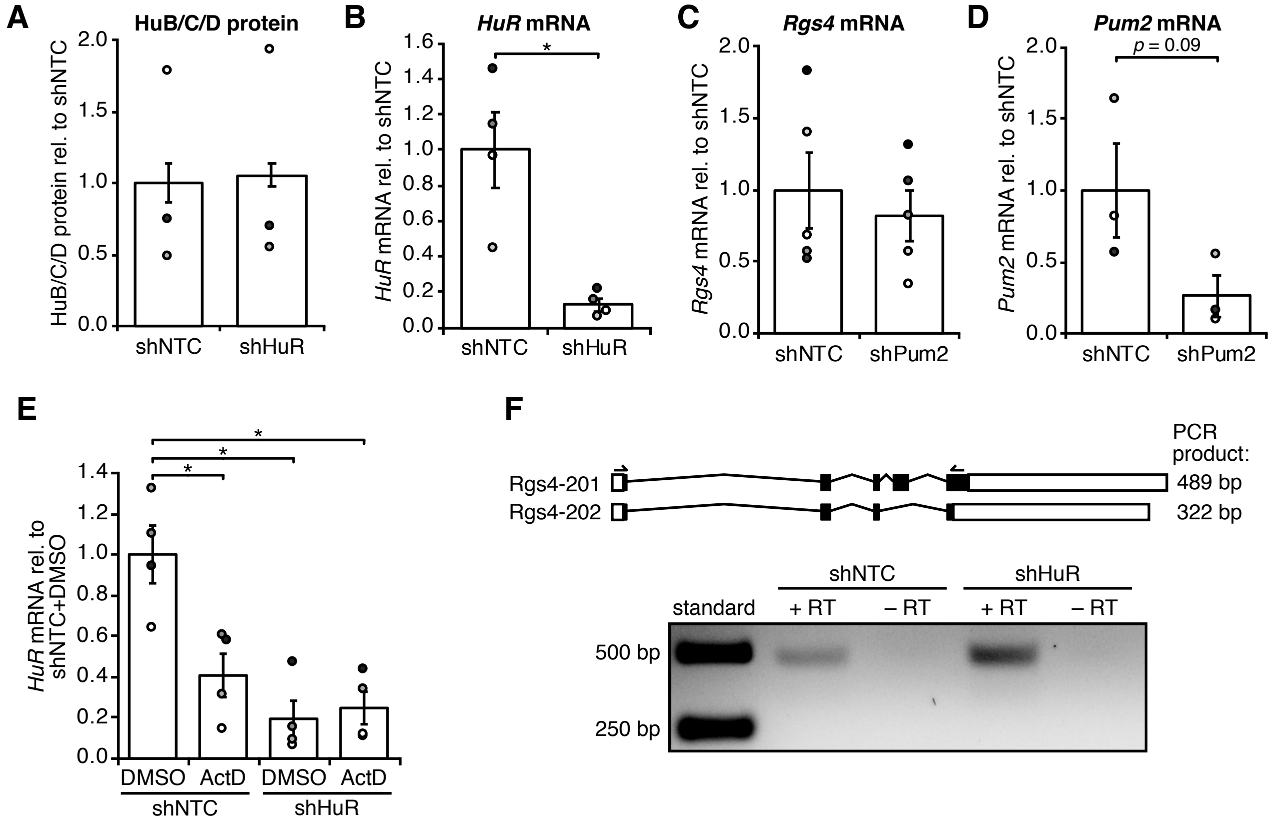

Supplementary Figure 1. Increase of *Rgs4* mRNA upon HuR knock-down is HuR specific. (A) Quantification of HuB/C/D western blot signal from 14 DIV cortical neurons transduced at 9+5 DIV with lentiviruses expressing shNTC or shHuR, normalized to shNTC. Paired Student's *t*-test. (B) Quantification of endogenous *HuR* mRNA by qRT-PCR from 14 DIV cortical neurons transduced at 9+5 DIV with lentiviruses expressing shNTC or shHuR, normalized to shNTC. Paired Student's *t*-test. (C) Quantification of endogenous *Rgs4* mRNA by qRT-PCR from 14 DIV cortical neurons transduced at 9+5 DIV with lentiviruses expressing shNTC or shPum2, normalized to shNTC. Paired Student's *t*-test. (D) Quantification of endogenous *Pum2* mRNA by qRT-PCR from 14 DIV cortical neurons transduced at 9+5 DIV with lentiviruses expressing shNTC or shPum2, normalized to shNTC. Paired Student's *t*-test. (E) Analysis of *HuR* mRNA stability in 14 DIV cortical neurons transduced at 9+5 DIV with lentiviruses expressing shNTC or shHuR and treated with DMSO or ActD for 90 min at 14 DIV. *HuR* mRNA levels were quantified by qRT-PCR and normalized to DMSO+shNTC. Paired Student's *t*-test. (F) RT-PCR with cDNA from 14 DIV rat cortical neurons transduced at 9+5 DIV with lentiviruses expressing shNTC or shHuR using primers detecting both annotated *mmuRgs4* splice isoforms (Ensemble, mm10). All error bars are SEM from ≥ 3 independent biological replicates; asterisks represent *p*-values (**p* < 0.05). KD knock-down; NTC non-targeting control; ActD Actinomycin D; bp base pair; DIV *days in vitro*.

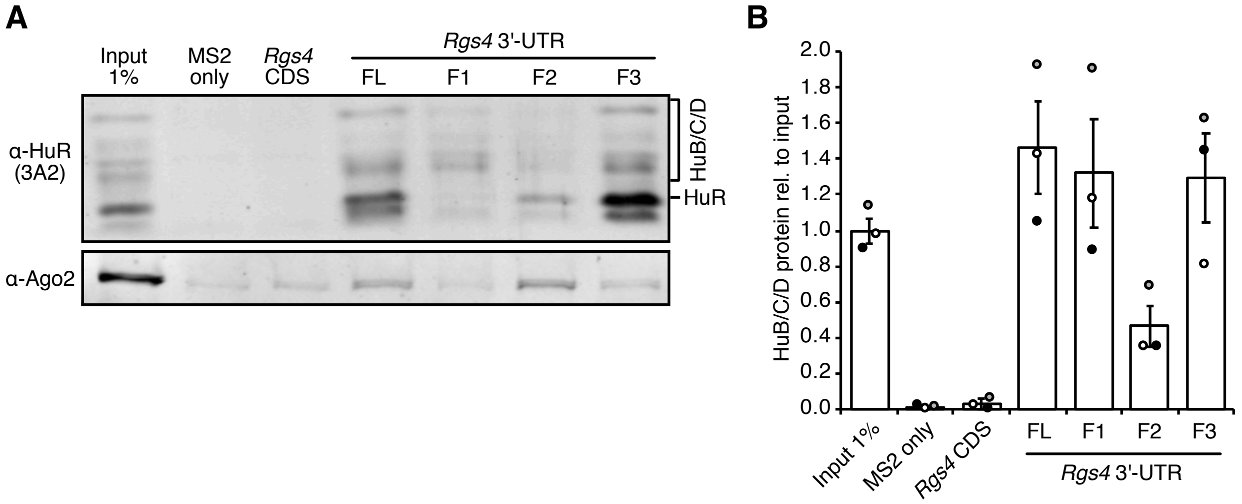

Supplementary Figure 2. HuB/C/D protein is less enriched in *Rgs4 in vitro* RNA purification. (A,B) Representative western blot against HuR (HuB/C/D) and Ago2 (A) and quantification (B) of HuB/C/D enrichment from adult rat cortex lysate in *in vitro* RNA affinity purification using 2xMS2 only, 2xMS2+*Rgs4* CDS, 2xMS2+*Rgs4* 3'-UTR, and different 2xMS2+*Rgs4* 3'-UTR fragments as bait RNA, normalized to input. All error bars are SEM from ≥ 3 independent biological replicates. WT wild type.

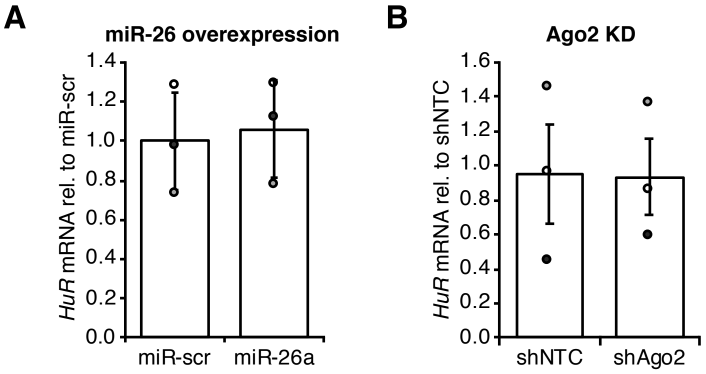

Supplementary Figure 3. *HuR* mRNA is not affected by miR-26 overexpression or Ago2 KD. (A) qRT-PCR analysis of endogenous *HuR* mRNA in 14 DIV cortical neurons transduced at 11+3 DIV with lentiviruses expressing miR-scr or miR-26a, normalized to miR-scr. (B) qRT-PCR analysis of endogenous *HuR* mRNA in 14 DIV cortical neurons transduced at 9+5 DIV with lentiviruses expressing shNTC or shAgo2, normalized to shNTC. Paired Student's *t*-test. All error bars are SEM from ≥ 3 independent biological replicates. KD knock-down; Scr scrambled; NTC non-targeting control; DIV *days in vitro*.

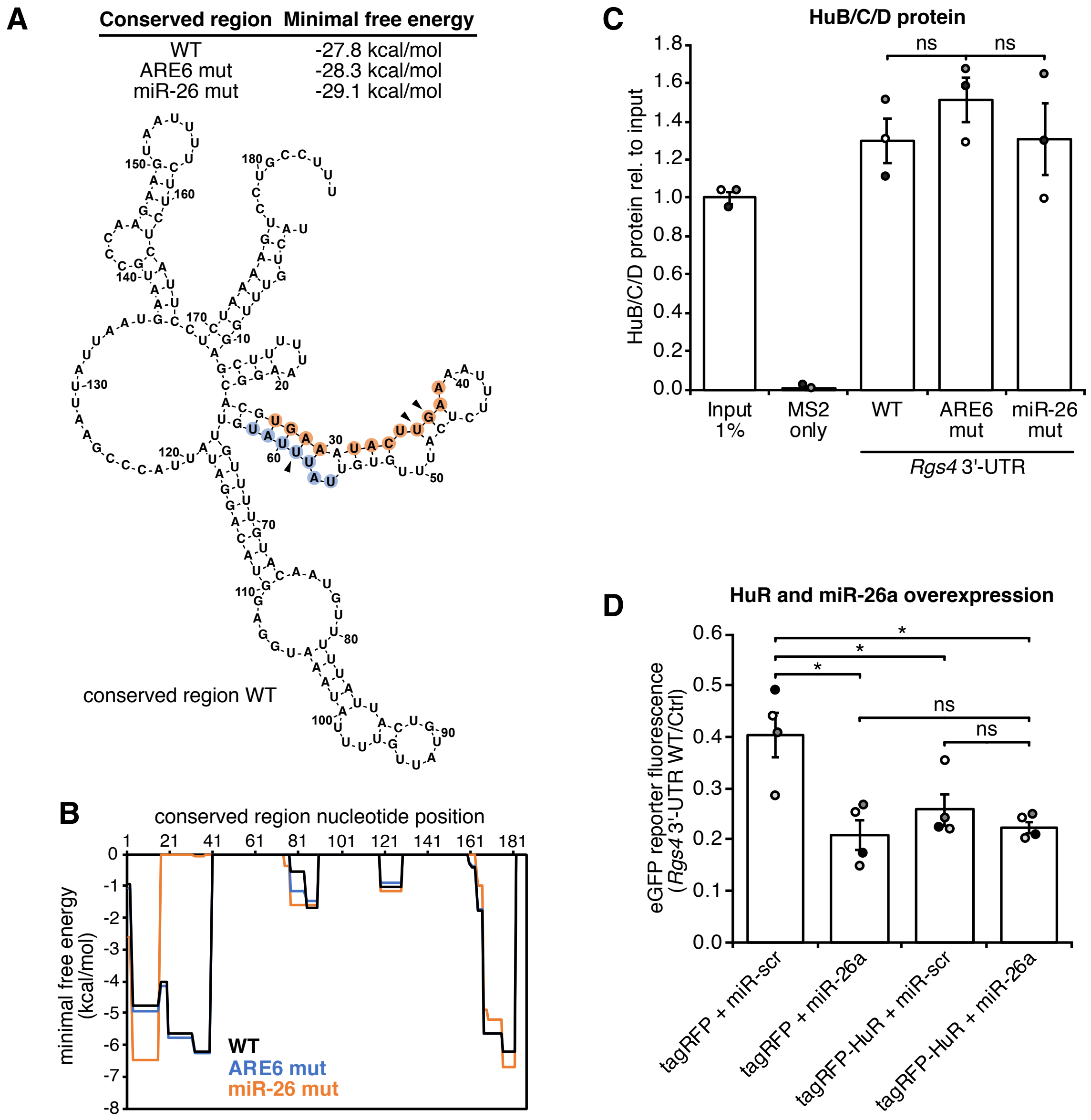

Supplementary Figure 4. HuB/C/D binding in *Rgs4 in vitro* RNA purification is not affected by ARE6 or miR-26 mutations. (A) Minimal free energies of predicted folding (RNAfold) of the conserved region of WT *Rgs4*-3'-UTR and ARE6 and miR-26 binding site mutants. (B) Minimal free energy of bound miR-26a to *Rgs4* conserved region calculated by IntaRNA for the *Rgs4* WT conserved region, and ARE6 and miR-26 binding site mutants. (C) Quantification of HuB/C/D enrichment from adult rat cortex lysate in *in vitro* RNA affinity purification using 2xMS2 only, 2xMS2+*Rgs4* 3'-UTR WT, 2xMS2+*Rgs4* 3'-UTR ARE6 mut and 2xMS2+*Rgs4* 3'-UTR miR-26 mut as bait RNA, normalized to input. Paired Student's *t*-test. (D) Quantification of eGFP fluorescence intensity in the cell body of hippocampal neurons at 15 DIV co-transfected at 14+1 DIV with eGFP-reporter and tagRFP or tagRFP-HuR and miR-scr or miR-26a. Ratio of eGFP reporter intensity between *Rgs4* 3'-UTR WT and Ctrl reporter is shown. Paired Student's *t*-test. All error bars are SEM from ≥ 3 independent biological replicates; asterisks represent *p*-values (**p* < 0.05). WT wild type; ARE AU-rich element; DIV *days in vitro*.

A

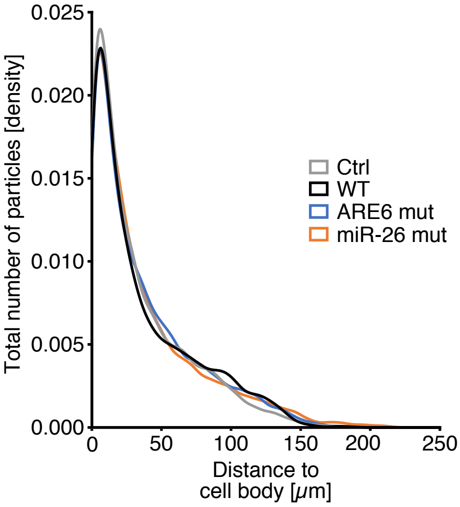

Supplementary Figure 5. Mutation of miR-26 and HuR binding sites does not change dendritic *Rgs4* RNA distribution. (A) Density plot of the total number of MS2 particles over distance from cell body from hippocampal neurons transfected with tdMCP-GFP and 128xMS2+Ctrl, 128xMS2+*Rgs4* 3'-UTR WT, ARE6 mut or miR-26 mut reporter mRNA at 14+1 DIV. ARE AU-rich element; WT wild type; tdMCP-GFP tandem MS2 coat protein fused to GFP; DIV *days in vitro*.
